# Supplementary material for: Association of the Gut Microbiota with the Host’s Health through an Analysis of Biochemical Markers, Dietary Estimation, and Microbial Composition
Source: Nutrients. 2022 Nov 23;14(23):4966. doi: 10.3390/nu14234966 (PMC9735734; doi:10.3390/nu14234966)
Supplement: Supplementary file 1 [file nutrients-14-04966-s001.zip › nutrients-1992125-supplementary.pdf]

Supplementary Materials

Figure S1. Biochemical markers and microbiota composition: Family and Genus.

Family			Genus		
Variables	Univariant test	Metagenome Seq	Variables	Univariant test	Metagenome Seq
AST			AST		
	Pasteurellaceae ↑	Pasteurellaceae ↑			Phascolarctobacterium ↑
	Oscillospiraceae ↓	Oscillospiraceae ↓			Haemophilus ↑
		Lactobacillaceae ↓			Lactobacillus ↓
		Rikenellaceae ↓			
		Porphyromonadaceae ↓			
HDL-c					
	Christensenellaceae ↑	Christensenellaceae ↑			
	Peptococcaceae ↓	Peptococcaceae ↓			
			Insulin		
					Phascolarctobacterium ↑
					Peptococcus ↑
					Butyricicoccus ↓

↑ Represents higher abundance of bacteria compared to the median of the individuals
↓ Represents lower abundance of bacteria compared to the median of the individuals

Figure S2. Nutritional markers and microbiota composition: Family.

Family		
Variables	Univariant test	Metagenome Seq
Protein intake		
	Unclassified clostridiales ↑	
Carbohydrate intake (%)		
		Enterobacteriaceae ↑
Fibre intake		
		Melainabacteriaceae ↓
Fruit intake		
		Enterobacteriaceae ↑
		Eubacteriaceae ↑
		Streptococcaceae ↑
Whole grains		
		Melainabacteriaceae ↓
Yogurt		
		Lactobacillaceae ↑
Fermented dairy		
		Lactobacillaceae ↑
Soda light		
		Clostridiaceae ↓
		Methanobacteriaceae ↓
		Dehalobacteriacesae ↓
Olive oil		
		Verrucomicrobiaceae ↑
Saturated fats		
		Acidaminococcaceae ↑

↑ Represents higher abundance of bacteria in the group of individuals above the median
↓ Represents lower abundance of bacterian in the group of individuals above the median

Figure S3. Nutritional markers and microbiota composition: Genus

Genus		
Variables	Univariant test	Metagenome Seq
Total energy		
		Paraprevotella ↑
Whole grains		
		Melainabacter ↓
Soda light		
		Ruminococcus ↑
		Eggerthella ↑
Olive oil		
		Phascolarctobacterium ↑
		Desulfovibrio ↓
Meat intake		
	Oscilospira ↓	Oscilospira ↓
		Phascolarctobacterium ↑
Cold meat		
	Butyricicoccus ↓	
Trans fat		
		Phascolarctobacterium ↑
Monounsaturated fat		
		Phascolarctobacterium ↑
Polyunsaturated fat		
		Butyricoccus ↑
Saturated fat		
		Phascolarctobacterium ↑
		Erysipelatoclostridium ↓

↑ Represents higher abundance of bacteria in the group of individuals above the median
↓ Represents lower abundance of bacterian in the group of individuals above the median
